# Comprehensive Intervention to Promote Healthy Lifestyles and Prevent Cardiometabolic Diseases in Low-Income School Children From Mexico: Protocol for the ESCOLARISANO Randomized Controlled Trial

**DOI:** 10.2196/82721

**Published:** 2026-05-06

**Authors:** Ana A Escárcega-Galaz, Elmer Enriquez-Rabago, Marcela Garcia-Moreno, Sara Leyva-Encinas, Cesar Robles-Aguilar, Mayra Arias-Gastelum, Gabriela Ulloa-Mercado, Pablo Gortares-Moroyoqui, Gabriela Maldonado-Ulloa, Juan F Hernandez-Chavez, Ana Renteria-Mexia

**Affiliations:** 1 Department of Biotechnology and Food Sciences, Instituto Tecnológico de Sonora, 5 de febrero 818 sur. Col. Centro, Ciudad Obregón, Sonora, 85000, Mexico, +526444109000 ext 1693; 2 Master in Science in Natural Resources, Department of Biotechnology and Food Sciences, Instituto Tecnológico de Sonora, Ciudad Obregón, Sonora, Mexico; 3 Faculty of Nutrition and Gastronomy Sciences, Universidad Autónoma de Sinaloa, Culiacán, Sinaloa, Mexico; 4 Department of Food and Nutrition, Universidad de Guadalajara, Guadalajara, Jalisco, Mexico; 5 Department of Agricultural and Veterinary Sciences, Instituto Tecnológico de Sonora, Ciudad Obregón, Sonora, Mexico

**Keywords:** healthy lifestyles, cardiometabolic disease prevention, Mexican schoolchildren, school intervention, childhood obesity

## Abstract

**Background:**

Obesity is a serious public health issue affecting children in a progressively alarming manner; thus, nutrition education and behavior change interventions during childhood are a priority. Eating and physical activity behaviors are mainly influenced by the environment; therefore, lifestyle changes are often successful when interventions are implemented in places such as the home and school. Schools are fundamental for ensuring the development of students’ personalities, skills, abilities, and long-term behaviors.

**Objective:**

This study aims to measure the effect of an intervention designed for low-income Mexican schoolchildren, focused on promoting healthy lifestyles for the prevention of obesity and cardiometabolic diseases.

**Methods:**

The study design is a randomized clinical trial (NCT05945862), with intervention groups paired with control groups of the same grade. Four elementary schools were selected based on district socioeconomic status. The study will be carried out for 1 year in four phases: (1) initial measurements, including anthropometry, blood pressure, psychosocial diagnosis, family medical history, and behavior variables, such as nutrition knowledge, dietary intake, sleep time and quality, and physical activity; (2) intervention implementation based on social cognitive theory, the behavior change wheel with the capability, opportunity, and motivation influencing behavior theoretical framework, and the transtheoretical theory, covering topics related to nutrition and healthy eating, child health, personal and sleep hygiene, physical activity, psychosocial well-being, and motivation; (3) postintervention measurements, including initial and behavioral measurements and intervention indicators; and (4) 6-month postintervention evaluation using the same initial and behavioral measures as in phase 1. Baseline differences by age, sex, socioeconomic status, and location will be analyzed using chi-square tests (qualitative variables) and analysis of covariance (quantitative variables). Multiple linear regression will test potential baseline associations between dependent variables (anthropometrics and blood pressure) and independent variables (diet, exercise, sleep time, family interaction, psychosocial well-being, and perception of childhood obesity). For comparing changes between the intervention and control groups at postintervention and at 6-month postintervention in anthropometrics, blood pressure, and behavioral variables, we will use multilevel mixed-effects regression models, given the hierarchical structure of participants nested within schools and the repeated measurements over time. SPSS and STATA software will be used with a significance level of *P*<.05.

**Results:**

From October 2023 to August 2024, a total of 451 participants were recruited from 4 different elementary schools in Sonora, México. As of February 2026, the data collected are in the process of being captured and analyzed.

**Conclusions:**

The protocol is a comprehensive program designed for schoolchildren in Mexico, intended to be an effective strategy for promoting healthy lifestyles and preventing cardiometabolic diseases. It is important to continuously adapt the intervention before implementation and to evaluate it to ensure its sustainability and expand its impact on other elementary schools, improving the health and well-being of schoolchildren.

## Introduction

Childhood obesity is a critical public health issue that has substantially increased worldwide [1,2]. Obesity is associated with metabolic and cardiovascular risks at all ages, but it is particularly risky at ages 2 to 19 years due to early complications [2,3]. According to UNICEF (United Nations International Children's Emergency Fund), at least 1 in 20 children under 5 years old has obesity; the prevalence in children over 5 years and adolescents increases to 1 in 3 [4]. Mexico ranks first in childhood obesity in the world, with 37.3% of schoolchildren and 41.1% of adolescents with overweight and obesity [5]. Lifestyle is a crucial risk factor for obesity; among obesogenic environments are unhealthy diets high in saturated fat, simple carbohydrates, and sugary drinks, as well as low physical activity (PA) with long periods of sitting [6,7]. Family factors, such as dysfunctional families and parents’ low educational levels, are also fundamental in childhood obesity. Lyons et al [8] reported that family discord influences children’s eating habits, with a higher risk of eating disorders later in life. Other studies indicated that mothers’ perceptions of the child’s body image are a risk factor for childhood obesity. Mothers of children with overweight or obesity tend to underestimate their children’s weight, representing a barrier to obesity diagnosis and treatment [9]. On the contrary, psychosocial well-being is a protective factor since it reduces emotional and behavioral disorders that could lead to obesity in children and adolescents [10].

Paradoxically, mild to moderate food insecurity and childhood malnutrition are also associated with a higher prevalence of obesity [11]. Low- and middle-income countries have the highest prevalence of malnutrition: undernutrition (11.5% growth retardation and 1.4% emaciation) and overweight (8.5%), placing Mexico in seventh place for child malnutrition in Latin America. Both obesity and malnutrition are risk factors for complications such as type 2 diabetes (T2D) and cardiovascular diseases, which in turn cause high mortality rates and decreased productive life [12]. Unfortunately, T2D in adolescence and childhood has increased, reaching a diagnostic prevalence between 0.68% and 1.2% [13]. Childhood obesity negatively affects cardiac and vascular structure and function; it triggers metabolic conditions such as insulin resistance, elevated serum uric acid, and metabolic syndrome, among others [14]. Estimates suggest that in the future, obesity-attributed coronary heart disease will increase by 5% to 16% [15].

Regarding lifestyle risk factors, studies have shown that 67.6% of schoolchildren and 46% of adolescents are sedentary, while 80.1% and 91.5% spend more than 2 hours in front of a screen. This leads to an increase in BMI and, therefore, a decrease in cardiovascular health [16]. On the contrary, a healthy diet is one of the main protective factors. Each region has traditional diets that, generally, are healthy and promote biodiversity and food security with low environmental impact. The traditional Mexican diet (TMexD) is a healthy and sustainable diet that promotes dietary diversity and encourages the consumption of local foods [17]. The TMexD includes plant foods such as fruits, vegetables, and whole grains, as well as animal-based foods, which provide fiber, vitamins, minerals, antioxidants, protein, and healthy fats. Promoting TMexD among Mexican schoolchildren, especially at ages 6 to 12 years, is crucial because they are more selective and demanding when it comes to consuming food [18]. Regarding PA in childhood, recommendations include at least 60 minutes per day in addition to limiting screen time to 2 hours per day or less [19]. Therefore, it is essential to implement behavior-change interventions promoting healthy diets and PA for childhood obesity prevention [20].

Multiple studies have concluded that it is easier to prevent obesity at early stages of life than in adulthood [18]. These interventions must take into account lifestyle settings and conditions such as those found in schools, homes, families, etc [21,22]. Proposals should focus on generating changes in eating habits, increasing PA, and raising awareness about the importance of healthy lifestyles among children and adolescents [15,23]. With a comprehensive intervention and its evaluation in low-income communities, the most unprotected social sectors will be prioritized [24].

Home and school are the most influential places for children. The school is considered a suitable and safe environment for programs promoting healthy lifestyles [25]. Studies have reported that school interventions focused on reducing obesity are successful because schoolchildren are at an age where healthy habits can be instilled and maintained in the long term [26,27]. On the other hand, parents as providers also influence nutrition during school years since children usually consume what is available at home. Furthermore, researchers focus on schools as a fundamental component to ensure the development of students’ personalities and habits, as well as to promote their multiple intelligences, skills, abilities, and behaviors. From this age forward, the educational center is a privileged environment to achieve a balance of these pillars that will last throughout life [28]. Thus, it is a priority for schools to promote healthy habits [21].

Globally, there are successful programs promoting healthy habits. The Diabetes Prevention Program is one of the first T2D preventive programs that has been adapted for use in school settings [29,30]. In Mexico, programs such as “Specific Action Program 2007-2012 School and Health,” “National Strategy for the Prevention and Control of Overweight, Obesity, and Diabetes,” and others have been developed for obesity and T2D prevention and have been taken as a basis for interventions in some Mexican elementary schools [31-33].

The present intervention protocol addresses childhood obesity in an integrative and unconventional manner, generating multifactorial knowledge about family behavior, individual factors, and the school environment. The aim of this protocol is to measure the effect of an intervention designed for low-income Mexican schoolchildren, focused on promoting healthy lifestyles for the prevention of obesity and cardiometabolic diseases. Specific aim 1 is to analyze whether baseline anthropometric and behavioral variables are associated with psychosocial well-being, family interaction or functionality, and perceptions of childhood obesity in the home environment. Specific aim 2 is to evaluate the effectiveness of the intervention in terms of retention, motivation to change, fidelity, and acceptability of the program by schoolchildren. Specific aim 3 focuses on evaluating changes in health behaviors at the end of the intervention and after 6 months in knowledge, diet quality, exercise time, and sleep time and quality.

## Methods

### Ethical Considerations

The protocol was approved by the Institutional Research Ethics Committee from the Technological Institute of Sonora (approval letter number 249). All procedures will be in accordance with the ethical standards of the ethics committee and with the Declaration of Helsinki. Prior to the study, written informed consent will be obtained from both the school principals and one parent or tutor, and informed assent will be obtained from the participants to authorize participation and the use of photographs and images. The intervention will be explained in detail, and participants will be informed that they are free to withdraw at any time, personal information will be kept confidential, and results will be presented after the complete intervention. The protocol was registered on ClinicalTrials.gov [34] with the identification number Protocol Record NCT05945862 (data registration: July 10, 2023), Healthy Lifestyle Promotion Program in Communities from the Yaqui Valley. Participants will be coded with a 3-digit number to keep their data confidential, and privacy will not be infringed. Identifying details will be omitted for the data analysis and publication processes.

### Study Design

**Figure 1.**
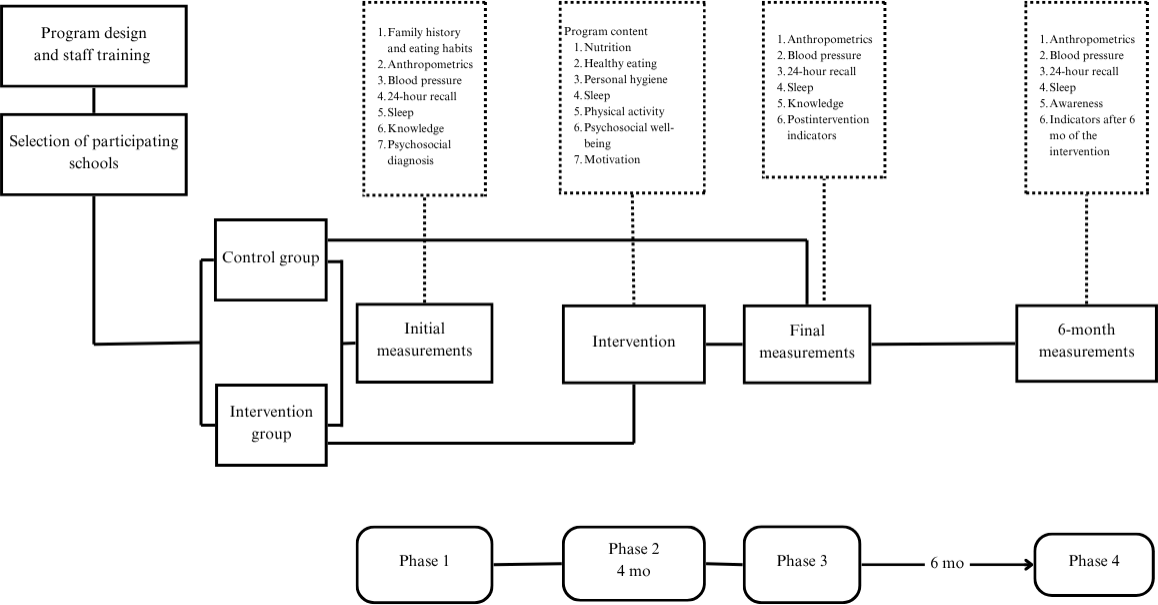
Study design for the ESCOLARISANO randomized controlled trial.

The study design is a randomized controlled trial in which school groups (classrooms) will be randomized either to the intervention or control arm. Baseline measurements will be completed before group assignment, reducing the potential for selection bias. The randomization sequence will be generated using a computerized random number generator (IBM SPSS Statistics version 21.0) by a researcher not involved in the study. Classroom groups will be paired within each school and randomly assigned (1:1) to the intervention or control arm. Allocation will be revealed by the independent researcher only after baseline measurements are completed and the intervention curriculum has been designed. The study will be conducted over 1 year in 4 phases. In the initial diagnostic phase (phase 1), baseline obesity risk factors will be measured in both control and intervention arms, assessing family and sociodemographic variables (family interaction, diet quality at home, body image perception [BIP], and psychosocial well-being) and schoolchildren’s health variables (anthropometry, diet, and blood pressure). Parents in both arms will receive a report on their child’s diagnosis, and in the control group, some recommendations will be added to improve their health and eating habits. Phase 2 consists of implementing the curriculum during 4 months in the intervention arm. Phase 3, at the end of the intervention, consists of the evaluation of results from the intervention arm in terms of knowledge acquisition, effectiveness, retention, motivation to change, fidelity, and acceptability by schoolchildren; in both arms, changes in knowledge, anthropometrics, blood pressure, and behavior of schoolchildren in terms of diet quality, exercise practice, and sleep quality will be measured. Finally, in phase 4, after 6 months of the intervention, sustained changes in anthropometrics and behavior (same measurements as phase 1) will be measured. The study design is shown in Figure 1. The program will allow for ongoing contact with participants, thereby promoting their retention and measuring immediate and medium-term outcomes. Participants in the control group will receive a summary version of the intervention in the next school year.

### Staff Training

Staff will be trained in data collection, intervention implementation, first aid, and childhood education techniques. Additional training is planned by community project experts to address different interventions.

### Selection of Schools

The districts and neighborhoods with the lowest economic resources in the region were identified using socioeconomic census data from the National Institute of Statistics and Geography (INEGI, for its acronym in Spanish), and 4 elementary schools were selected according to logistical feasibility and resources: 1 in Loma de Bácum (representing the rural area) and 3 in Ciudad Obregón, Sonora, México (representing the urban area). An interview will be held with the school principals to present the proposal, and those who agree will be asked for written authorization. Within each school, an equal number of groups (classrooms paired by grade) will be selected for the intervention and control arms, allowing for approximate matching of participants by sex and age in each arm. This approach allows us to offer the program to at least half of the students in each school and to implement the intervention in as many schools as possible according to available resources.

The number of classrooms per school is determined by the existing school structure and logistical feasibility. Across the 4 schools, a total of 24 classrooms will be included (12 in the intervention arm and 12 in the control arm), with varying numbers of students (participants) per classroom, since student grouping is pre-established by the Ministry of Education at the beginning of the school year.

### Participants

The inclusion criteria are being students aged 6 to 13 years enrolled in the selected schools, with availability to complete the intervention; residents of either Ciudad Obregón and/or Valle del Yaqui, Sonora; and having informed consent from a parent or guardian. The exclusion criteria are having any physical, metabolic, or mental illness that prevents them from carrying out the intervention or undergoing a health intervention program or diet, exercise, lifestyle, and/or therapeutic treatment in the 3 months prior to the study. Data collection instruments, questionnaires, and intervention activities were tailored for lower (children aged 6‐9 years, up to grade 3) and upper (children older than 9 years in grades 4 to 6) elementary school groups. For all school groups, a staff member acted as a support interviewer in the application of instruments and questionnaires.

### Program Promotion

A promotional campaign with flyers and posters will be conducted 1 month prior to the intervention both inside the schools and in nearby strategic locations. A face-to-face meeting will be held with parents where the program will be presented.

### Health Diagnosis

#### Family Medical History

The family medical history will be obtained before the intervention based on the Mexican Official Standard NOM-004-SSA3-2012 [35].

#### Anthropometric Measurements

Anthropometrics will be carried out in triplicate based on the methodology established by the World Health Organization [36] and Centers for Disease Control and Prevention [37].

##### Weight

A TANITA brand scale with integrated bioimpedance (model BC-533; Tanita Corporation of America, Inc) will be placed on a firm, flat surface. Participants must be barefoot, with their feet centrally positioned on the scale, arms at their sides, wearing as little clothing as possible, and avoiding carrying heavy objects (eg, belts, coins).

##### Height

A SECA 213 stadiometer (SECA Deutschland, Medical Scales and Measuring Systems) will be used. Participants must be barefoot and without any objects on their head (such as hairpins, buns, high hairstyles, and caps), standing with feet centered on the stadiometer and heels together at a 45° angle. The head, back, calves, heels, and buttocks must be in contact with the instrument, with arms falling naturally at their sides and the head in the Frankfurt plane [38]. The midsagittal line of the body must coincide with the midline of the instrument.

##### BMI and BMI Percentile

BMI will be calculated with the equation: BMI=(weight in kg)/(height in m^2^). BMI percentile will be calculated using the Centers for Disease Control and Prevention (2000) graphs, classifying participants as underweight, normal weight, overweight, or obese.

##### Waist Circumference Percentile and Waist-to-Height Ratio

Waist circumference (cm) will be measured with a Lufkin brand tape at the umbilicus level and classified as central obesity when it is above the 90th percentile [39], following the methodology proposed for Mexican schoolchildren and adolescents [40]. For the waist-to-height ratio, waist circumference will be divided by height (both in cm). Classification will be carried out according to the cutoff points proposed by Marrodan et al [41], with categories of overweight in boys with a waist-to-height ratio ≥0.48 and in girls ≥0.47; and obesity in boys ≥0.51 and in girls ≥0.50.

##### Body Fat (%) and Fat Mass Index

The measurement will be carried out using bioimpedance integrated into the TANITA scale (model BC-533; Tanita Corporation of America, Inc.), and participants will be asked to stand barefoot and in the Frankfurt plane, removing any metal and heavy objects that could interfere with the measurement. Body fat percentile will be calculated using the corresponding cutoff points [42]. Fat mass index, calculated by dividing kilograms of fat by height in m², will be classified into normal (≤) or overweight or obese categories (>) with limits of 7.76 in girls and 4.58 in boys from 8 years of age [43].

### Blood Pressure

The procedure will follow the NOM-030-SSA2-2009 [44] using a portable digital OMRON HEM-7120 blood pressure monitor. Measurements will be taken in triplicate on the left arm. Participants must be seated with back support, and measurements should be taken after 5 minutes of rest, leaving at least 2 minutes between repetitions.

### Family and Psychosocial Diagnosis

#### Family Interaction

Family interaction will be evaluated with the APGAR (Adaptability, Partnership, Growth, Affection, and Resolve) questionnaire, measuring family cohesion between parents and participants [45]. The response scale has the following measures: almost never, sometimes, and almost always. Scores (0 to 10) will be classified as follows: 0 to 2 indicates severe dysfunction; 3 to 6 indicates mild dysfunction; and greater than 6 indicates good functionality [46]. This questionnaire has been used in adolescents, schoolchildren, parents, and guardians and has been validated in its Spanish version [45].

#### Psychosocial Well-Being

We will have 2 versions of the Strengths and Difficulties Questionnaire adapted according to the age category: 4 to 9 years and 10 to 12 years [47]. It consists of 38 questions inquiring about emotional well-being, behavior, and social skills, including their relationships with themselves and others. This questionnaire has been used in school-aged children and adolescents in its validated Spanish version [48,49].

#### Body Image Perception

BIP will be assessed according to the methodology adapted by Collins et al [50] and validated for schoolchildren and adolescents. According to the authors, no cultural adaptation was required, as the instrument is based on visual figures and contains minimal textual content beyond instructions. It consists of 7 anatomical figures increasing in body volume, separately for male and female participants, representing different BMI percentiles. Participants will choose the figure that most closely matches their own silhouette, as well as the ideal silhouette they would like to resemble. Body image dissatisfaction will be assessed using a Likert scale questionnaire with response options of satisfactory, slightly satisfactory, and not at all satisfactory. Agreement of conformity will be assessed using the methodology adapted from Marrodán et al [51], comparing real BMI with perceived BMI, grouping participants into 3 categories: (1) values <−4 to −2, overestimate their BMI; (2) values between −2 and +2, perceive themselves as they are; (3) values between +2 and +4, underestimate their BMI. The feel-actual inconsistency index (feel-perceived weight status minus actual-real weight status) will be classified as follows: (1) score 0, consistency in the perception of weight status; (2) positive score, overvalued weight status; (3) negative score, undervalued weight status [52].

#### Family Adherence to the TMexD

Adherence to the TMexD will be estimated with a list of 15 groups of typical Mexican foods proposed by Valerino-Perea et al [53]. A score is assigned to each food, summing the corresponding points if the food was consumed and no points (0) if not consumed, with 21 points being the highest possible score. This instrument was originally developed in Spanish and specifically designed for the Mexican population, applied in national survey data (Encuesta Nacional de Salud y Nutrición).

### Design of the ESCOLARISANO Program Curriculum

**Figure 2.**
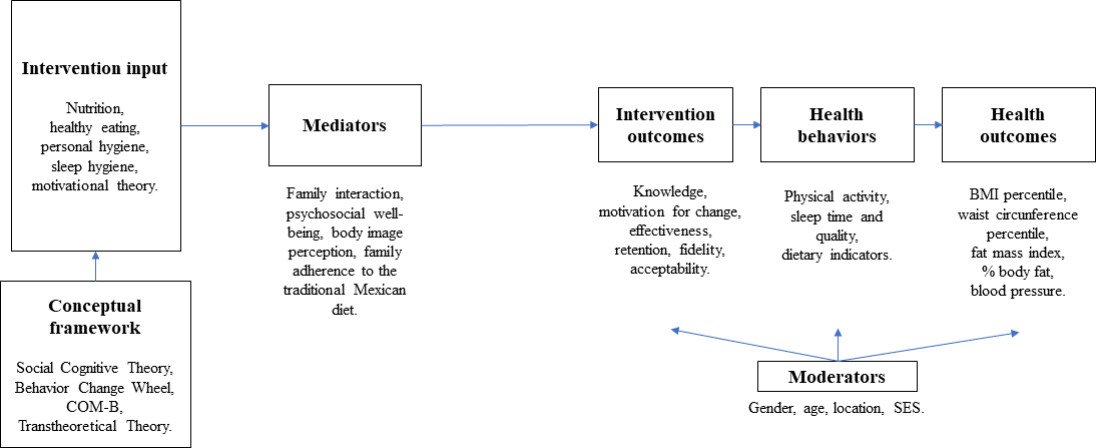
Conceptual model for the ESCOLARISANO randomized controlled trial. COM-B: capability, opportunity, and motivation influencing behavior; SES: socioeconomic status.

The curriculum was based on social cognitive theory (SCT), the behavior change wheel (BCW) with the COM-B theoretical framework (capability, opportunity, and motivation influencing behavior), and the transtheoretical theory (TTT), which have been used in public health programs for disease prevention and health promotion [54-56]. Program content includes recommendations from official health and childhood institutions [57] for the prevention of malnutrition, food insecurity, and cardiometabolic diseases [58]. Nutrition education and cooking workshops, adapted to the regional, cultural, and social context of participants, will be delivered in two 1-hour weekly sessions. One additional 1-hour weekly PA session will reinforce the official Ministry of Education PA curriculum. A conceptual model, shown in Figure 2 was proposed to explain the intervention and the relationship among its components, the thematic content, and the variables involved. The operationalization of study constructs through intervention components is detailed in Table 1. The intervention will be implemented over 4 months, covering the topics and activities outlined in Table 2.

**Table 1.** Operationalization of study constructs through intervention components.

COM-B^a^ components and SCT^b^ constructs	TTM^c^ stages targeted	Behavior change mechanisms	Intervention components
Psychological capability			
Behavioral capability	Precontemplation → contemplation	Increased knowledge and practical skills required to perform the behavior.	Nutrition education: educational sessions on healthy eating, food label reading, water consumption, and the role of breakfast.
Observational learning	Contemplation → preparation	Learning through positive role models.	Guided demonstrations (healthy lunchbox preparation), visual examples, and group-based activities.
Outcome expectations	Contemplation	Cognitive restructuring regarding short- and long-term benefits.	Guided discussions on the health benefits of healthy eating, physical activity, and sleep.
Self-regulation (emotional regulation)	Preparation → action	Emotion identification and regulation.	Emotion awareness: learning to recognize emotions, applying emotion regulation strategies, and addressing body shaming.
Physical capability			
Behavioral capability	Action	Development of physical skills to perform the behavior.	Physical activity promotion: guided and supervised physical activity practice.
Reflective motivation			
Self-regulation	Preparation → action	Planning, monitoring, and adjustment of behavior; integration of multiple healthy behaviors.	Goal setting, habit tracking, follow-up, and feedback.
Self-efficacy	Preparation	Increased perceived confidence to engage in the behavior.	Achievable goal setting and feedback.
Outcome expectations	Action → maintenance	Conscious evaluation of behavioral consequences.	Guided reflection on perceived changes in well-being and energy levels.
Automatic motivation			
Reinforcement	Action → maintenance	Habit strengthening through positive reinforcement.	Verbal recognition, feedback, and social reinforcement.
Social opportunity			
Observational learning	All stages	Social normalization of healthy behaviors.	Group activities and participatory dynamics.

a COM-B: capability, opportunity, and motivation influencing behavior.

b SCT: social cognitive theory.

c TTM: transtheoretical model.

**Table 2.** Thematic content of the intervention.

Phases	Description	Instruments/Indicators/Activities
0. Staff training and recruitment	Staff training on techniques and methodologies for anthropometric and dietary assessment, etc. Preparation and standardization of tools, instruments, materials, questionnaires, and data recording sheets. Recruitment logistics and curriculum design.	Advertising campaign for the intervention. Printing of questionnaires and/or paper formats. Practical rehearsal for staff, specifically on anthropometry and completing questionnaires and/or instruments.
1. Baseline measurements	Baseline measurements.	Application of baseline questionnaires and recalls: informed consent, family medical history, anthropometrics, blood pressure, knowledge, body image perception, family interaction (APGAR)^a^, psychosocial well-being (SDQ^b^), adherence to the traditional Mexican diet, 24h-R^c^, sleep time, and quality.
2. Intervention curriculum	Topics: promotion of a healthy lifestyle, physical exercise, health education, child nutrition and health, emotions and mental health, personal hygiene, cooking workshops, prevention of cardiometabolic diseases (overweight, obesity, diabetes, and hypertension).	Activities: putting on a t-shirt; healthy eating; exercising, getting active, and moving one’s body; recognizing one’s emotions and mental health; preparing a healthy lunch; sip by sip; cleansing, loving and taking care of one’s body; reducing screen time; bedtime habits; healthy breakfast; reading nutrition labels; cooking workshop 1: preparing fruit salad with yogurt, cooking workshop 2: preparing refreshing drinks, cooking workshop 3: preparing banana popsicles with peanut butter; comprehensive healthy living.
3. Final measurements (immediately after intervention)	Measurements. Data analysis: data capture, review, cleaning, and modeling.	Anthropometrics and blood pressure. Questionnaires and recalls (same as baseline). Assessment of intervention indicators: effectiveness, retention, motivation to change, viability, fidelity, feasibility, and acceptability. Data analysis using SPSS software.
4. Final measurements (6 months postintervention)	Measurements. Data analysis: data capture, review, cleaning, and modeling.	Anthropometrics and blood pressure. Questionnaires and recalls (same as baseline). Data analysis using SPSS software.

a Family APGAR (Adaptability, Partnership, Growth, Affection, and Resolve): a questionnaire used to assess family functioning and the level of satisfaction family members have with their relationships.

b SDQ: Strengths and Difficulties Questionnaire.

c 24h-R: 24-hour recall.

Participants will be encouraged to engage in a variety of free PA activities at school and home [19]. Among the activities are walks, runs, ball games, etc. It is crucial to include this diversity of topics and activities because it is a multidisciplinary program characterized by addressing the problem comprehensively. Participant attendance will be recorded, and evidence of the activities for each topic will be kept. Communication with teachers and parents or guardians via WhatsApp and in person at schools will be available at all times to address questions regarding the research, children’s progress, behavior, and diagnoses or findings.

### Behavioral Indicators (Before and After the Intervention)

#### 24-Hour Recall

Food intake will be assessed preintervention and postintervention in children in grades 4 to 6 using 2 nonconsecutive 24-hour recalls (24h-R), with the United States Department of Agriculture Automated Multiple-Pass Method [59]. The procedure consists of conducting an individual interview by a dietitian staff member and recording, on paper, the participant’s total food consumption during the day prior to the interview (from the time the individual woke up until the time they went to bed), ensuring that all foods and beverages are included. During the interview, key questions will be asked to stimulate the participants’ memory. To estimate the participant’s daily nutrient intake, recalls will be processed on the ASA24 site by the dietitian staff member, and for dishes not listed on the ASA24 platform, the ingredients will be individually itemized and recorded on the website.

#### Indicators of Diet Quality

The following dietary quality indicators will be estimated to examine their association with cardiometabolic risk factors at baseline and to assess changes in eating behaviors after the intervention.

##### Healthy Eating Index

The Healthy Eating Index (HEI) 2024 will be estimated [60]. The overall HEI score will be calculated using the following classification: (1) optimal, with an HEI score greater than or equal to 81; (2) requires improvement, with an HEI score between 51 and 80; (3) poor, with an HEI score less than or equal to 50 [61].

##### NOVA System

The classification of foods by NOVA groups will be carried out into the following categories: NOVA 1: nonperishable or minimally processed foods; NOVA 2: processed culinary ingredients; NOVA 3: processed foods; NOVA 4: ultra-processed foods (UPFs) [62].

##### Dietary Components Related to Cardiometabolic Risk

Dietary components related to cardiometabolic risk include daily consumption of risky dietary components, such as total and added sugars, sodium, and saturated fat, among others.

### Physical Activity

The 3-day physical activity recall assesses the time spent on different types of activities rated as light, moderate, intense, or very intense, recorded in periods of 30 minutes from when the interviewee got up in the morning until they went to sleep. Activities are assigned a metabolic equivalent of task (MET) level (moderate-to-vigorous physical activity ≥3 METs; vigorous physical activity ≥6 METs), and the summary scores are tallied as total METs per day [63]. This questionnaire has been validated in Latin American children and adolescents [64] and will be administered to participants from grades 4 to 6 [65].

### Sleep Hygiene

Sleep time and quality will be assessed using the 24-hour sleep questionnaire applied on 3 different days. The questionnaire consists of 12 questions covering the start and end of sleep (bedtime, waking time, and getting-up time), sleep characteristics (latency, number of sleep interruptions, sleep duration), subjective perception (how you felt upon waking, sleep quality), and comparison with usual habits (perceived amount of sleep). Information is collected using the ASA24 sleep module [66].

### Intervention Indicators

#### Preintervention Feasibility and Viability

The feasibility and viability assessment will be carried out prior to the intervention using checklists. We will assess whether the proposal is feasible in the specific context of the school, adapting the program to the conditions of each school, and meeting technical, economic, and human resource requirements based on the number of participants per school.

#### Assessment of Knowledge and Motivation for Change

Acquired knowledge, motivation for change, and satisfaction will be evaluated through a pretest and posttest. For motivation and willingness to change behavior [67], confidence and the importance attributed to the change are analyzed through 2 questions: “What level of confidence do you feel in being able to make this change?” on a scale of 1 (zero confidence) to 10 (extreme confidence); and “How important is it for you to make this change?” where 1 indicates little importance and 10 indicates the utmost importance [68].

#### Effectiveness

Results obtained in the experimental arm will be compared with those of the control arm to analyze the significant differences in behavioral and health outcomes between them. This comparison will provide a more robust perspective on the real impact of the intervention on the habits and health of the participants [69].

#### Retention

The retention rate will assess the proportion of participants who complete the study compared to those who were initially recruited. A high retention rate is key to ensuring the validity of the results. It is calculated by dividing the number of people who completed the intervention by the total number of individuals enrolled at the start [70].

#### Fidelity

Fidelity involves assessing participants’ responses as a result of the practices used to ensure that the responses reliably and validly represent the purpose of the intervention. It will be evaluated through checklists in each session with the following criteria: attention, positive attitude, active participation, work in the sessions, and attendance lists [27].

#### Acceptability

Acceptability will be assessed by measuring perception and satisfaction at the end of the intervention, analyzing aspects such as effectiveness, motivation, barriers, and limitations, which will allow for obtaining feedback to improve the design of future studies. To evaluate the social aspect of the program’s acceptability, participants will be given a survey with both open-ended and multiple-choice sections using a Likert scale [32,71-73].

### Statistical Analysis

The normal distribution of the data will be verified using skewness, kurtosis, and Kolmogorov-Smirnov tests. Quantitative data with nonnormal distribution will be mathematically transformed for parametric analysis, or alternatively, nonparametric analysis will be used. Means and SDs will be calculated for continuous variables and percentages for categorical variables. To test age, sex, socioeconomic status, and location baseline differences, we will use chi-square tests for qualitative demographic variables (eg, socioeconomic level, family medical history, sport practice, etc) and analysis of covariance for quantitative variables (eg, anthropometrics, dietary intakes, HEI, TMexD, sleep time, BIP, family interaction, and psychosocial well-being). To evaluate specific aim 1, potential baseline associations of dependent variables (eg, anthropometrics and blood pressure) with independent behavioral variables (eg, dietary intakes, exercise and sleep time, family interaction, psychosocial well-being, and perception of childhood obesity) will be tested using multiple linear regression, starting with univariate regression (*P*<.2 and biological plausibility). Models will be built by a combination of univariate analysis and forward stepwise model selection using *P*≤.05 and biological plausibility. Preliminary models will be assessed for possible interactions (*P*≤.1) between sex and other variables included in the model, for collinearity (variance inflation factor<10), and for linear regression assumptions (linearity, normality, and homoscedasticity) using residual plots. Specific aim 2 will use mixed methods (qualitative and quantitative) to analyze results in terms of retention, fidelity, and acceptability of the intervention by children and school administrators. Specific aim 3 will compare the changes between the experimental arms (intervention vs control) from baseline to postintervention and to the 6-month follow-up in anthropometrics, blood pressure, HEI, dietary intakes, PA, sleep time, and knowledge. Primary analyses will follow the intention-to-treat principle. Given the hierarchical structure of the data (students nested within classrooms and classrooms within schools) and the repeated measurements over time (baseline, postintervention, and 6-month follow-up), intervention effects will be evaluated using multilevel mixed-effects regression models. Due to the cluster-based allocation, the intraclass correlation will be considered, potential attrition will be monitored, and all analyses will account for clustering to provide valid inferences. Models will include fixed effects for study arm, time, and the arm×time interaction, and random effects for classroom and school to account for intracluster correlation. Baseline values of the outcomes and covariates will be included as appropriate. Effect estimates with 95% CIs will be reported. We will examine model assumptions and fit indices. SPSS (IBM SPSS Statistics version 21.0) and STATA (StataCorp version 14.0) software will be used, with a *P* value <.05 for all statistical analyses.

## Results

From October 2023 to August 2024, a total of 451 participants were recruited from 4 different elementary schools in Sonora, México. As of February 2026, the collected data are in the process of analysis and interpretation. Table 3 presents the current status of each stage of the protocol as of February 2026.

**Table 3.** Current status of the research protocol.

	Participating schools			
	Nueva Creación (26EPR0373T^a^)	Miguel Hidalgo (26DPR1013P^a^)	Héroe de Nacozari (26DPR0251J^a^)	Gral. Plutarco Elías Calles (26DPR0250K^a^)
Training and program design	✓	✓	✓	✓
Selection of participating schools	✓	✓	✓	✓
Sample size, n	173	117	86	75
Initial measurements	✓	✓	✓	✓
Intervention	✓	✓	✓	✓
Final measurements	✓	✓	✓	✓
Follow-up (measurements at 6 months)	✓	✓	✓	✓
Date capture	✓	✓	✓	✓
Data analysis	✓	✓		
Data interpretation				

a Work Center Code of the primary school.

## Discussion

Lifestyle interventions in early life are critical to reducing future disease risk and health care costs. During recent years, most of the research has been toward adult behavior [23], but recently, successful programs have been adapted to children and adolescents [21,33], representing an opportunity to extend their benefits to youth. For a greater probability of success, participant and setting characteristics should be accounted for. Places where children spend most of their time are school and home; schools represent a structured environment for the implementation of planned activities, fostering knowledge acquisition and sustainable behavioral changes. Within schools, a broader population is reached, and participants support each other, sharing experiences [25,26,74].

Two main biological models have been proposed to explain obesity: the carbohydrate-insulin model [64] and the energy balance model [75], the latter reinforced by current evidence on the high energy contribution of UPFs. Our intervention is primarily based on the carbohydrate-insulin framework, which posits that diets high in rapidly absorbed carbohydrates—often from UPFs, also high in energy, saturated fat, and sodium—promote hyperinsulinemia and adiposity. Accordingly, the intervention focused on improving carbohydrate quality and overall dietary patterns by reducing UPF consumption, along with structured PA to improve insulin sensitivity and body composition, and therefore reduce obesity. Secondary cardiometabolic outcomes were examined within this same framework, given the influence of diet and PA on metabolic and vascular health. Taken together, it is hypothesized that these behavioral changes improve insulin sensitivity, inflammation, and vascular function, thereby reducing cardiometabolic risk.

Theories such as the SCT, the TTT, and the BCW have been used as a framework in obesity studies. The SCT characterizes behavior, with self-efficacy as the greatest motivator and mediator of actions, highlighting the importance of setting goals, social and professional support [54]. Activities are planned from informing to putting practices into action to create a healthy lifestyle through education, motivation, persuasion, development of capacities, modeling, and training. The BCW indicates that for a change in behavior, capacity, opportunity, and motivation must be present [56]. With respect to the TTT, it assumes that behavior change develops in staggered stages, and while the intervention is implemented, participants may progress through stages as they increase awareness, motivation, and engagement in health-related behaviors [55]. In the present study, the transtheoretical model was used as part of the conceptual framework, and although participants were not formally classified into discrete stages, intervention activities were designed to target key processes across stages of change, progressing from awareness and outcome expectations (precontemplation/contemplation) to self-efficacy and planning (preparation), to practice (action), and finally to reinforcement and self-monitoring (action/maintenance).

Within the intervention, components align with constructs in Table 1, targeting cognitive, psychological, and physical capability through knowledge acquisition, skills training, and observational learning, while reflective and autonomous motivation were addressed by enhancing self-efficacy, outcome expectations, self-regulation, and reinforcement processes; social opportunity was incorporated through group-based activities, reducing contextual barriers to healthy behaviors. As summarized in Table 1, theoretical constructs from the SCT, COM-B, and transtheoretical model were operationalized using psychosocial and behavioral indicators assessed, in most cases, with validated instruments.

The study’s conceptual model in Figure 2 posits that the intervention influences outcomes indirectly through internal processes and individual factors. Mediating variables such as BIP and adherence to the TMexD, among others, strengthen personal and social resources, thereby facilitating the adoption and maintenance of healthy habits. Moderating variables (such as sex and location, etc) shape the context in which these changes occur and potentially influence how individuals interpret, assimilate, and respond to the intervention, ultimately modulating observed outcomes.

Regarding child nutrition, Bujtor et al [76] identified that unhealthy dietary patterns high in fat and sugary drinks were associated with impaired body composition and inflammatory biochemical parameters (ie, inflammatory cytokines, C-reactive protein, total cholesterol, triglycerides, glucose, and insulin) [77]. Likewise, healthy dietary patterns (ie, those rich in fruits and vegetables and low in UPF and sugary beverages) should be promoted from early childhood, given that children tend to exhibit greater food selectivity. With respect to PA patterns, studies reported that almost 50% of children and adolescents usually spend more time in sedentary activities (ie, screen time), consuming more sugary and salty foods, with an increase in weight and body fat [15,78]. On the contrary, an active PA pattern is associated with improvements in adiposity [79], self-esteem, mental health [80], quality of life, cognitive abilities, academic performance, etc [81]. Even low-intensity PA promotes favorable health changes in children and young people with obesity [1].

As reported by Heggmen and Zahl [82], children from rural communities have a higher risk of overweight, obesity, and unhealthy habits. Some causes include limited access to healthy foods, less space availability for PA, and less nutrition and health education. However, children from urban communities are also at risk of obesity due to their social environment: globalization, modernization, cultural changes, and low economic conditions [74]. This proposal includes participants from rural and urban communities and schoolchildren from mestizo and indigenous origins, ensuring equity, cultural relevance, and social justice, reducing the gap and improving external validity due to participants’ diversity [25-27].

Among the indicators of effectiveness are efficacy, acceptance, knowledge acquisition, motivation, etc [70-73]. Knowledge acquisition assesses participants’ learning process; by working on the learning constructs, a change in long-term habits could be achieved [24,78]. Other critical variables are motivation and willingness to change, adherence to the intervention, retention, and dropout rates, which will be assessed by directed questions and statistical techniques (ie, intention-to-treat analysis). Fidelity consists of evaluating the practices used to ensure that the intervention is reliably and validly applied and that changes after the sessions can be attributed to the intervention and not to external factors, reducing the likelihood of errors and bias. Regarding participants’ understanding and involvement, attention, positive attitude, active participation, class work, attendance lists, etc, will be assessed using checklists. Supervision instruments monitoring the organization, planning, execution, and use of the protocols and the curriculum manual are also important [83,84]. Acceptability indicates how participants perceive the intervention; if it is not well accepted, it can generate a lack of commitment and reduced effectiveness. Once participants take ownership of the intervention, they are more likely to maintain their practices even after the intervention ends [71,72].

Among the strengths are its reach and accessibility, as it was conducted in schools where children spend a lot of time, representing a realistic and ideal environment for a large number of participants, with a lower probability of withdrawal. School age represents a critical growth period in the formation of lasting habits, and children can be multiplying intervention agents in their families. Another strength is the involvement of rural and urban schools, which promotes equity and reduces the disparity in cardiometabolic risk between both communities. The experimental design using school groups as units optimizes resources and allows for a larger number of schools to be intervened. Another advantage is the comprehensive approach for assessing family habits such as adherence to the TMexD and psychosocial variables. Individual variables such as diet, sleep, and PA times are fundamental in lifestyle habits, and evaluating the intervention in terms of feasibility, viability, retention, and acceptability allows measurement of the sustainability of acquired skills over time. Regarding the use of repeated-measures ANOVA, this is a robust tool in this type of study evaluating participants at multiple times.

Among the limitations, classrooms were randomized within schools, which, while not the optimal design for this type of study, enabled us to offer the intervention to more participants in each school. Logistical and funding constraints limit a second-year waiting-list control design, leading us to prioritize implementation across multiple schools. School schedules (eg, recess, arrival, and dismissal) are structured to minimize interaction between classrooms, reducing the likelihood of contamination. Nevertheless, we acknowledge that some contamination may occur and will address this with appropriate statistical analyses. Another limitation is the relatively low sample size for this kind of study, since this type of design requires a larger number of participants and the use of robust statistical methods. However, our research design is comparable to similar studies [74].

The age of participants may also limit their responses, since younger schoolchildren find it difficult to express themselves and remember data (ie, quantity of food and time of PA). This could be improved through greater interaction between staff and participants to build trust and facilitate recall of daily activities, enhancing the accuracy of responses. Regarding the self-reported estimates of PA and dietary intake, they depend on short- and long-term memory, showing greater variability compared to other objective measures [85,86]. However, the 3-day physical activity recall and the 24h-R are validated instruments widely used in the child population [87,88]. The use of few repetitions of the 24h-R does not ensure the measurement of between- and within-person variability; however, administering more repetitions could increase the challenges inherent in data collection in children and adolescents, who may have difficulty sharing information about their behaviors and may experience mental burden and stress [89]. Another limitation is that other important social actors in the student’s life, such as parents, will not be involved; however, the involvement of teachers, and especially classmates, is possible. The curriculum implementation depends on the time available in the school curriculum itself; however, if the intervention begins alongside the school year, it optimizes time and makes it easier to complete it. Another opportunity for future studies is to include other crucial variables such as quality of life and self-esteem, which are important at this stage. Concerning multilevel models, these require larger sample sizes, involve more complex assumptions, and may be sensitive to model misspecification. However, these are well suited for group-randomized trials, as they account for data grouping, provide valid standard errors, accommodate missing or unbalanced data, and allow the simultaneous examination of individual- and group-level effects, thereby improving statistical efficiency and inference.

In conclusion, the proposed protocol represents a comprehensive program and an effective and feasible strategy to promote healthy lifestyles and prevent cardiometabolic diseases, addressing low-income schoolchildren in Mexico. It is important to continuously evaluate and adapt the intervention to ensure sustainability and expand its impact to other schools. The implementation of this program can significantly contribute to improving children’s health and well-being by fostering positive habits from an early age and reducing the risk of obesity and cardiometabolic complications.

## Supplementary material

10.2196/82721 Checklist 1 SPIRIT checklist.

## Abbreviations

24h-R: 24-hour recall
APGAR: Adaptability, Partnership, Growth, Affection, and Resolve
BCW: behavior change wheel
BIP: body image perception
COM-B: capability, opportunity, and motivation influencing behavior
HEI: Healthy Eating Index
INEGI: National Institute of Statistics and Geography
MET: metabolic equivalent of task
PA: physical activity
SCT: social cognitive theory
SDQ: Strengths and Difficulties Questionnaire
T2D: type 2 diabetes
TMexD: traditional Mexican diet
TTT: transtheoretical theory
UNICEF: United Nations International Children's Emergency Fund
UPF: ultra-processed food
